# Characterization of La_2_O_3_ Nanoparticles and Their Effects on Bacteria, Vero and MG63 Cells, and Zebrafish Development

**DOI:** 10.3390/bioengineering12090995

**Published:** 2025-09-18

**Authors:** Jugal Kishore, Tharaka Srinatha Dunuwilla, Venkatagiri Krishnamoorthy Bupesh Raja, Stanley Abraham Louis, Lokesh Kumar Boopathy, Durai Saravanan, Mzia Zhvania, Manoj Gupta

**Affiliations:** 1 Faculty of Medicine, Georgian National University SEU, Tbilisi 0144, Georgia; 2 Laboratory of Neuron Ultrastructure and Nanoarchitecture, Ivane Beritashvili Center of Experimental Biomedicine, Tbilisi 0112, Georgia; mzia_zhvania@iliauni.edu.ge; 3 School of Medicine, Alte University, Tbilisi 0177, Georgia; tharaka.dunuwila@hotmail.com; 4 Department of Automobile Engineering, School of Mechanical Engineering, Sathyabama Institute of Science and Technology, Chennai 600119, India; bupesh.v.k@gmail.com; 5 Centre for Ocean Research, National Facility for Coastal and Marine Research, Sathyabama Research Park, Sathyabama Institute of Science and Technology, Chennai 600119, India; sabraham.cor@sathyabama.ac.in; 6 Department of Biochemistry, JR Medical College and Hospital, Thindivanam 604302, India; lokeshkumarunom@gmail.com; 7 Centre for Laboratory Animal Technology and Research, Sathyabama Research Park, Sathyabama Institute of Science and Technology, Chennai 600119, India; snegapoorvam@yahoo.com; 8 School of Natural Sciences and Medicine, Ilia State University, Tbilisi 0162, Georgia; 9 Department of Mechanical Engineering, National University of Singapore, Singapore 117576, Singapore

**Keywords:** Vero cells, MG63 cell lines, rare-earth nanoparticles, developmental toxicity, metal oxide nanoparticles

## Abstract

This study reports, for the first time, lanthanum oxide (La_2_O_3_) nanoparticles (NPs) that simultaneously suppress osteosarcoma MG63 cell proliferation and promote normal Vero cell viability, a dual effect not previously documented for La_2_O_3_ or similar metal oxide NPs. Physico-chemical characterization revealed a unique needle-like morphology, cubic crystallinity, and dispersion stability in DMSO without acidic dispersants, properties that can influence cellular uptake, ROS modulation, and biocompatibility. Comprehensive characterization (fluorescence spectroscopy, particle size/zeta potential, Raman, XRD, TGA, ATR-FTIR, and TEM) confirmed structural stability and surface chemistry relevant to biological interactions.La_2_O_3_ NPs exhibited broad-spectrum antibacterial activity (Gram-positive *Streptococcus pyogenes*, *Bacillus cereus*; Gram-negative *Escherichia coli*, *Pseudomonas aeruginosa*) and strong enzymatic/non-enzymatic antioxidant capacity, supporting potential use in implant coatings and infection control. MTT assays demonstrated dose-dependent cytotoxicity in MG63 cells, with enhanced proliferation in Vero cells. In zebrafish embryos, developmental toxicity assays yielded an LC_50_ of 2.6 mg/mL higher (less toxic) than values reported for Ag NPs (~0.3–1 mg/mL) with normal development at lower concentrations and dose-dependent malformations (e.g., impaired somite formation and skeletal deformities) at higher doses. Collectively, these findings position La_2_O_3_ NPs as a multifunctional platform for oncology and regenerative medicine, uniquely combining selective anticancer activity, normal cell support, antimicrobial and antioxidant functions, and a defined developmental safety margin.

## 1. Introduction

Biomedical research continually seeks novel materials with improved therapeutic performance. Lanthanum oxide (La_2_O_3_), a rare-earth metal oxide, has attracted interest for its cytotoxic and growth-inhibitory properties in various biomedical applications. Such nanoparticles, by virtue of their small size, large surface area, and unique bio-interactivity, enable applications in drug delivery, imaging, cancer therapy, and antimicrobial treatments. Nanoparticles are broadly classified into metal-based (e.g., silver, gold, and zinc oxide), metal-oxide-based (e.g., La_2_O_3_ and cerium oxide), non-metal (e.g., carbon nanotubes and graphene), and polymeric types. They may be synthesized via physical, chemical, or biological (“green”) methods, each influencing particle properties and biocompatibility. Physical methods allow precise control, chemical methods are cost-effective and scalable, and biological routes are eco-friendly with potential biocompatibility advantages [1,2]. La_2_O_3_ has been applied as an MRI contrast agent, a hypophosphatemic agent in kidney dialysis, and as a luminescent probe for cellular imaging [3,4,5,6]. La_2_O_3_ NPs have been shown to sensitize glioblastoma cells to radiation and temozolomide, and have potential in photodynamic therapy, radiation therapy, targeted drug/gene delivery, biosensing, and bioimaging [7,8,9]. Lanthanum can modulate redox balance and influence reactive oxygen species (ROS) homeostasis, with potential implications for health and survival [10]. Despite these promising properties, the interactions between La_2_O_3_ NPs and different cellular systems remain incompletely understood, with conflicting reports on cytotoxicity and biocompatibility [11,12,13,14,15,16]. In comparison, other metal-based nanoparticles such as silver (Ag), gold (Au), zinc oxide (ZnO), and titanium dioxide (TiO_2_) have been extensively studied for antimicrobial, anticancer, and antioxidant activities. However, their dose-dependent cytotoxicity often limits clinical translation, underscoring the need for alternatives with wider therapeutic windows. Non-metal-based nanoparticles (e.g., graphene oxide, carbon nanotubes, and polymeric systems) tend to be more biocompatible but often lack the strong intrinsic bioactivity of metallic systems.La_2_O_3_ NPs combine high stability, redox activity, and rare-earth-element-specific biological interactions, making them promising therapeutic candidates, yet their cell-type-specific cytotoxicity remains underexplored.

Studying La_2_O_3_ cytotoxicity in both normal (Vero) and cancerous (MG63) cells is critical to determining their therapeutic potential and selectivity. Vero cells have shown susceptibility to various toxicants, including T-2 toxin, rare-earth nanoparticles, and microcystin-LR [7,13,17]. Such findings underscore the need to understand cytotoxic mechanisms and differential responses across cell types. Similarly, amine-functionalized amorphous silica nanostructures—sharing certain properties with La_2_O_3_—have increased cytotoxicity in Vero cells [18]. These parallels suggest that La_2_O_3_ NPs could exert cytotoxic effects on both Vero and MG63 cells, warranting comparative evaluation.

Conversely, oxygen-deficient La_2_O_3−x_ NPs showed no toxicity in non-malignant human keratinocytes, suggesting possible biocompatibility [16]. However, mesoporous SiO_2_-based nanocomposites containing La_2_O_3_ were toxic to multiple cell lines [19], reflecting variability in biological responses.La_2_O_3_ has also demonstrated cytotoxicity toward freshwater bacterial isolates [19] and exhibited phytotoxicity in cucumber plants [20]. Collectively, these findings indicate that La_2_O_3_ NP cytotoxicity and biocompatibility are influenced by particle size, surface charge, and environmental context. Prior work attributes these effects to the inherently small particle size of La_2_O_3_ NPs, which increases surface area, reactivity, and environmental sensitivity [21].

The cytotoxicity of La_2_O_3_ NPs is thought to involve oxidative stress and DNA damage, mechanisms that also motivate investigation of their antioxidant potential. Similar mechanisms have been reported for other metal oxide NPs, including Al_2_O_3_ and α-MnO_2_, which have induced cytotoxicity through comparable pathways [11,22,23,24]. In glioblastoma cells, La_2_O_3_ NPs enhanced radiation and temozolomide efficacy via increased apoptosis and ROS generation [7]. The release of cytotoxic proteins in LAK cells through both Ca^2+^-dependent and Ca^2+^-independent mechanisms suggests possible pathways for La_2_O_3_-induced cytotoxicity [25]. Additionally, endoplasmic reticulum stress and autophagy have been implicated in La_2_O_3_-related toxicity in Vero-E6 and HepG2 cells [17]. Conversely, oxygen-deficient La_2_O_3−x_ NPs have shown ROS scavenging properties [16], suggesting that under certain conditions they may protect rather than damage cells [16].

The potential antioxidant role of La_2_O_3_ has been examined in Vero cells, where oxygen-deficient variants synthesized by spray pyrolysis scavenged ROS and were biocompatible with non-malignant keratinocytes [16]. This suggests that La_2_O_3_ may have a protective effect on cells, including Vero cells, by reducing oxidative stress. Several studies have highlighted that different compounds can produce diverse effects across cell lines [26,27,28,29]. However, systematic evaluation of La_2_O_3_ effects on Vero cells under controlled conditions remains lacking, which this study addresses.

Zebrafish have emerged as a valuable vertebrate model bridging invertebrates (worms and flies) and mammals (mice and humans) for developmental and toxicological studies [30]. For example, zebrafish embryos exposed to Ag NPs exhibited reduced heart rate, impaired sensory function, and increased mortality with developmental abnormalities [31]. Such findings raise concerns over nanoparticle exposure risks to human health and ecosystems, underscoring the need for developmental toxicity testing [32]. Zebrafish studies have advanced understanding of nanoparticle-induced developmental malformations and genetic alterations in embryos [33]. Recent work suggests La_2_O_3_ NPs may serve as targeted drug carriers in aquatic models, reducing required dosages and minimizing side effects [34].

Despite these advances, no prior study has shown La_2_O_3_ NPs that promote normal cell proliferation, selectively suppress cancer cell growth, and maintain low developmental toxicity. Here, we report La_2_O_3_ NPs with atypical needle-like morphology and cubic crystallinity, stably dispersed in DMSO without acidic additives, facilitating direct biological assessment. This combination of physicochemical stability and dual biological activity establishes these NPs as a novel platform for targeted cancer therapy and regenerative medicine.

## 2. Materials and Methods

### 2.1. Materials

The lanthanum oxide (La_2_O_3_) NPs used in this study were of the size 80–100 nm with a purity of 99.9% (Nano Research Lab, Jharkhand, India). The Vero cell line and MG 63 cell line were obtained from the National Centre for Cell Sciences (NCCS), Pune, India. 

### 2.2. Methods

#### 2.2.1. Preparation of La_2_O_3_ NPs Dispersion

**Figure 1:**
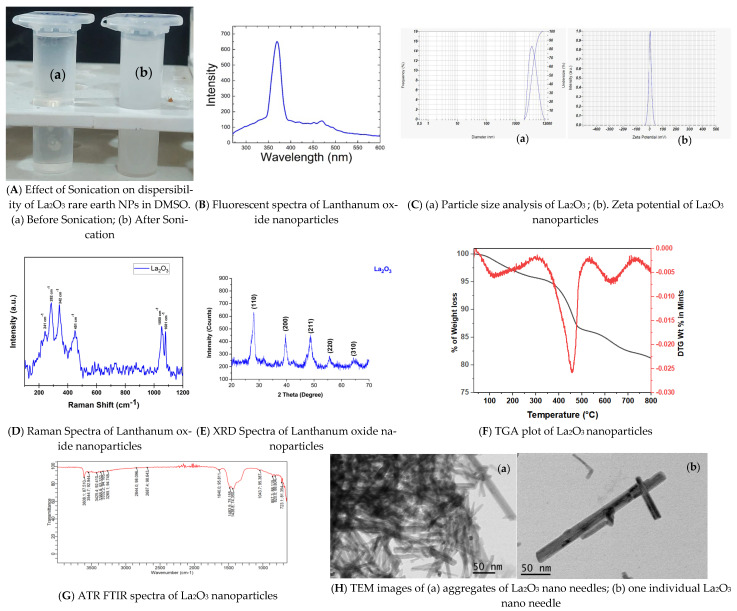
Physico-Chemical Characterizations.

To increase the solubility, about 1% of La_2_O_3_ *w*/*v* was dispersed in dimethyl sulfoxide(DMSO) and sonicated at 50 Hz for 5 min. The nanoparticles were dispersed and homogenized in the solvent as shown in Figure 1A. The solution was preserved at 4 °C for further characterization and for performing bioactivity assays.

#### 2.2.2. Characterization Using Fluorescent Spectrophotometer

The La_2_O_3_ NP solution was brought to room temperature and subjected for fluorescent spectrofluorometry analysis (Shimadzu-RF5301pc, Kyoto, Japan), and its excitation wavelength was observed in the range of 200 nm to 600 nm. The blank of DMSO was used as the base reference.

#### 2.2.3. Characterization of Particle Size and Zeta Potential

The particle size distribution and zeta potential were analysed using a particle size analyser (Horiba nanopartica SZ100V2, Kyoto, Japan).

#### 2.2.4. Characterization of La_2_O_3_NPs by Raman Spectroscopy

Raman spectra were obtained using an inVia Micro Raman spectrophotometer (Renishaw, New Mills, UK) equipped with different objectives such as 5×, 20×, 50×, and 100×. However, the samples were analysed using a 50× objective lens. The laser wavelength used to acquire Raman spectra was 785 nm with a power of 3 mW. The spectra were obtained in the range of 100–1500 cm^−1^ with a laser exposure time of 30 s. Wire 5.5 software was used to process the obtained spectra. 

#### 2.2.5. Characterization of La_2_O_3_ NPs by X-Ray Diffraction (XRD)

La_2_O_3_ NPs used in the present study were subjected to X-ray diffraction (XRD) analysis. XRD was operated with Cu Kα1 radiations using ARL EQUINOX 3000 (Ecublens, Switzerland) to analyseLa_2_O_3_ NPs.

#### 2.2.6. Characterization of La_2_O_3_ NPs by Thermogravimetric Analysis (TGA)

TGA of La_2_O_3_NPs was recorded in the range of 0 °C to 800 °C. 

#### 2.2.7. Characterization of La_2_O_3_ NPs by Attenuated Total Reflectance Fourier Transform Infrared (ATR-FTIR) Spectroscopy

The ATR-FTIR was carried out using Agilent MicroLab (method file: Default.a2m) with a resolution of 8 cm^−1^ with 32 sample scans and 32 background scans. The wavenumber range was 4000–650 cm^−1^ with Happ–Genzel as the apodization function. The sample was prepared and placed on the ATR crystal. Background scans were recorded before sample measurement. The sample was scanned under the same conditions as the background, i.e., 32 scans were averaged to reduce noise. The FTIR spectrum was generated, showing wavenumber (cm^−1^) vs. intensity, and peaks were identified and recorded.

#### 2.2.8. Characterization of La_2_O_3_ NPs by Transmission Electron Microscopy (TEM)

The TEM analysis was carried out by coating the La_2_O_3_ solution onto the copper grid, and its shape, surface morphology, and particle size were determined. An acceleration voltage of 200 kV was used to acquire TEM images with a JEOL3010 transmission electron microscope (Tokyo, Japan).

### 2.3. Antibacterial Assay

Resazurin microtitre assay is widely used for compounds that have less solubility. In this study, a redox indicator was used to evaluate the minimum inhibitory concentration of La_2_O_3_ NPs with varying concentrations of 2.5 mg, 1.25 mg, 0.625 mg, 0.312 mg, 0.156 mg, 0.078 mg, 0.039 mg, and 0.019 mg against selected clinical pathogens viz. *Streptococcus pyogenes (ATCC 19615)*, *Bacillus cereus (ATCC 11778)*, *Escherichia coli (ATCC 25922)*, and *Pseudomonas aeruginosa (ATCC 27853)*. To perform the assay, 50 µL of standard HiMedia™ Nutrient Broth (M002) sterile nutrient broth, composed of peptone (5 g/L), beef extract (3 g/L), and sodium chloride (5 g/L), prepared in distilled water and sterilized by autoclaving, was taken in each well separately for each concentration in sterile 96-well plates, and to each corresponding well, 50 µL of serially diluted concentration of La_2_O_3_ NPs was incorporated. Later, 3.3× strength of iso-sensitized broth was added to each well. Afterwards, 10 µL of 24 h grown suspension of each bacterial pathogen was added into each well separately, and in a separate well 10 µL of azithromycin as a positive control, and in two separate wells 10 µL of each DMSO and nutrient broth without clinical pathogen inoculum were maintained as a negative control. Observation of change in colour from blue or purple to pink was noted as a positive result, and finally the growth of the pathogens was evaluated by measuring the absorbance at 660 nm using a multi-mode reader [35].

### 2.4. Antioxidant Assay

#### 2.4.1. Enzymatic Glutathione Reductase Activity of La_2_O_3_ NPs

Glutathione reductase enzymatic antioxidant activity was measured following the method proposed by Goldberg and Spooner [36]. The sample was diluted to 50 µg/mL, 100 µg/mL, 200 µg/mL, 400 µg/mL, and 800 µg/mL. To a set of clean test tubes, 50 µL of diluted sample was added, and 1.0 mL of potassium phosphate buffer was homogenized with 100 mM potassium phosphate (pH 7.0 with 1 mM EDTA), 100 µL of GSSG (glutathione disulfide), and 100 µL of 2 mM Nicotinamide Adenine Dinucleotide Phosphate Hydrogen (NADPH). The antioxidant activity was measured at 340 nm in a UV Visible Spectrophotometer (Schimadzu, Tokyo, Japan). The unit of glutathione reductase activity was expressed as the amount of enzyme required to reduce 1 µmol of GSSG, where GSSG is the oxidized form of glutathione (GSH), per minute, which is equivalent to the oxidation of 1 µM of NADPH per minute.

#### 2.4.2. Non-Enzymatic Antioxidant Activity by 1,1-Diphenyl-2-picrylhydrazyl (DPPH) Assay

This assay is the measure of antioxidant activity of the molecules capable of scavenging the DPPH radicals, which was determined by the spectrophotometric method. To a clean set of test tubes, 100 µL of diluted samples (50 µg/mL, 100 µg/mL, 200 µg/mL, 400 µg/mL, and 800 µg/mL) along with 3.7 mL of methanol were added; this was followed by 200 µL of DPPH reagent, which was added into all the test samples, and 100 µL of Butylated Hydroxy Toluene (BHT) was maintained as a standard. A separate 3.8 mL of methanol was used as a blank. All the tubes (blank, standard, and test) were incubated at room temperature in a dark condition for 30 min, and the scavenging activity was measured spectrophotometrically at 517 nm [37]. The DPPH radical scavenging activity (RSA) was calculated using the following formula [38].

(1) $$\mathrm{DPPH}\;\mathrm{activity}\;(\%)=\frac{\left(Absorbance\;of\;blank)-(Absorbance\;of\;test\right)}{\left(Absorbance\;of\;blank\right)}\times 100$$

### 2.5. Cell Viability Assays (In Vitro Assay for Cytotoxicity Activity: MTT Assay)

The cells were maintained in Dulbecco’s Modified Eagle’s Medium (DMEM) supplemented with 10% foetal bovine serum (FBS), penicillin (100 U/mL), and streptomycin (100 μg/mL) in a humidified atmosphere of 5% CO_2_ at 37 °C. 

The Vero cell line and MG 63 cell line were exposed to the La_2_O_3_ NPs with various concentrations, and the cell viability assay was carried out. Cells (1 × 10^5^/well) were plated in 24-well plates and incubated in 37 °C with a 5% CO_2_ condition. After the cell reaches the confluence, the various concentrations of the samples were added and incubated for 24 h. After incubation, the samples were removed from the well and washed with phosphate-buffered saline (pH 7.4) or DMEM without serum. In this study, 100 µL/well (5 mg/mL) of 0.5% 3-(4,5-dimethyl-2-thiazolyl)-2,5-diphenyl--tetrazolium bromide (MTT) was added and incubated for 4 h [39]. After incubation, 1 mL of dimethyl sulfoxide (DMSO) was added to all the wells. The absorbance at 570 nm was measured with a UV spectrophotometer using DMSO as the blank. Measurements were performed, and the concentration required for a 50% inhibitory concentration (IC50) was determined graphically. The cell line morphological changes were studied via optical microscopy (OM), with images taken at 100× magnification.

The cell viability percentage was calculated using the following formula [40]:

% Cell viability = A570 of treated cells/A570 of control cells × 100(2)

A stands for absorbance, 570 nm.

Graphs are plotted using the percentage of cell viability along the *Y*-axis and the concentration of the sample along the *X*-axis. The cell control and sample control are included in each assay to compare with the full cell viability assessments.

### 2.6. Zebrafish Husbandry and Embryo Culture

The zebrafish used in this study were housed at the Centre for Laboratory Animal Technology and Research (CCSEA Approved) at Sathyabama University, Tamil Nadu, India. Adult zebrafish were maintained in a controlled environment at a temperature of 28 °C, pH 7 ± 0.2, and a 14:10 light–dark cycle. They were fed a diet of dry shrimp flakes twice daily and live brine shrimp once daily. To ensure optimal husbandry, the Organisation for Economic Co-operation and Development (OECD) guidelines for zebrafish care were strictly adhered to. Water quality parameters, including pH, were monitored daily and maintained within the recommended range of 6.8–7.5. The experimental tanks were inspected daily to assess zebrafish behaviour and overall health. For breeding, adult zebrafish were housed separately by sex prior to pairing. Fertilized embryos were collected and placed in Petri dishes containing methylene blue and growth medium [41]. The graph was plotted using GraphPad Prism Version 8.

#### 2.6.1. Teratogenicity Assay

To assess the impact of La_2_O_3_ NPs on zebrafish embryo development, a controlled experiment was conducted. Embryos were exposed to various concentrations of La_2_O_3_ NPs (namely, 1, 1.4, 1.8, 2.2, and 2.6 mg/mL) within an E3 medium environment. A control group, devoid of NP exposure, was included for comparison. Each experimental condition was replicated twice, with three replicate samples per experiment. The experiment was carried out under sterile conditions at a constant temperature of 28 °C. The growth and development of the embryos were monitored periodically, and the embryos were then examined for morphological abnormalities including defects in organ formation, such as heart, brain, or skeletal abnormalities, reduced body size and weight, delayed hatching, or slower developmental rates. The morphology of the embryos was visualized under a Leica stereomicroscope at 500 µm to analyse the developmental stages from 0 h to 72 h.

#### 2.6.2. Percentage Mortality

Mortality was assessed visually according to OECD guidelines. Embryos were considered dead if they displayed no heartbeat, a white precipitate at the bottom of the well, lack of movement, and a release of unpleasant odour.

(3) $$\%\;\mathrm{Mortality}=\frac{Number\;of\;dead\;embryos}{Total\;embryos}\times 100$$

#### 2.6.3. Percentage Hatchability

Hatchability is assessed by the successful transformation of embryos into larvae that exhibit normal locomotor behaviour.

(4) $$\%\;\mathrm{Hatchability}=\frac{Number\;of\;hatched\;embryos}{Total\;embryos}\times 100$$

#### 2.6.4. Percentage Heartbeat

To assess the effects of La_2_O_3_ NPs on heart function, zebrafish embryos were exposed for 96 h. Heart rate was determined by manually counting heartbeats over a 15-s period and multiplying this value by four to obtain the beats per minute (bpm).

## 3. Results and Discussion

### 3.1. Physico-Chemical Characterizations

#### 3.1.1. Solubility of La_2_O_3_ NPs

Sonication at 50 Hz for 5 min enhanced dispersion of La_2_O_3_ NPs in DMSO, shown in Figure 1A, producing a stable suspension suitable for biological assays. This approach improves bioactivity potential while eliminating the need for acidic dispersants commonly used for other metal oxides [42].

#### 3.1.2. Characterization of La_2_O_3_ NPs Using a Fluorescent Spectrophotometer

Fluorescence spectra shown in Figure 1B revealed a dominant 370 nm emission band, corresponding to band-edge transitions in La_2_O_3_, indicative of an intrinsic electronic structure. A weaker 420 nm violet emission, consistent with defect-related transitions from oxygen vacancies and cation sites following Judd–Ofelt transition rules, was also observed [43,44]. Additional peaks, including a prominent 468 nm feature, were present alongside minor emissions [45]. These combined bands suggest luminescence in La_2_O_3_ is governed by crystallite size, morphology, and defect chemistry, factors controlled during synthesis. DMSO controls showed no measurable emission, confirming La_2_O_3_ NPs as the sole signal source. Prior work attributes the 420 nm peak to the absence of electrons in the La^3+^ 4f shell, as crystalline La_2_O_3_ cannot be emitted from the inner 4f states [46]. These emissions are largely attributed to cation vacancy defects. Specifically, the 420 nm band likely reflects transitions between induced defects, the O 2p band, and the near band edge [45]. Variations in reported emission maxima likely arise from synthesis-dependent differences in crystallite size, defect density, and measurement conditions. For example, a sonication-assisted hydrothermal synthesis yielded La_2_O_3_ NPs (~41 nm) with dominant 645 nm red emission, reflecting deeper defect states [47], in contrast to our near-UV/violet profile [48]. In contrast, our synthesis yielded near-UV/violet emissions (~370–420 nm) as primary features. These findings underscore the sensitivity of La_2_O_3_ NPs luminescence to synthesis conditions, defect structures, and measurement parameters.

#### 3.1.3. Particle Size Analysis and Zeta Potential of La_2_O_3_ NPs

Dynamic light scattering indicated an average particle size of ~3906.6 nm, reflecting substantial agglomeration of crystalline La_2_O_3_ NPs during measurement. Such aggregation likely results from moisture adsorption, promoting clump formation during sample preparation. This increased effective size diminishes quantum confinement effects, potentially altering optical properties. In smaller NPs, strong quantum confinement typically widens the band gap. Agglomeration reduces this effect, narrowing the band gap [47]. Overall, samples displayed broad size distributions due to agglomeration. The zeta potential was +5.8 mV, indicative of moderate colloidal stability. This surface charge influences dispersion stability and may correlate with biological interactions and toxicity [49]. The particle analysis graph and zeta potential graph are shown in Figure 1C(a) and Figure 1C(b), respectively.

#### 3.1.4. Characterization of La_2_O_3_NPs by Raman Spectroscopy

Raman spectroscopy probed phonon vibrational modes to complement XRD-derived structural data. Spectra indicated La_2_O_3_ crystallized in mixed cubic and hexagonal phases. Figure 1D shows the Raman spectrum of as-prepared La_2_O_3_ NPs, acquired using a 785 nm excitation laser. Peaks were observed at ~241, 282, 342, 451, 1050, and 1081 cm^−1^. The 342 and 451 cm^−1^ peaks correspond to La–O bending and stretching modes. Peaks at 241, 282, 342, and 451 cm^−1^ represent A_1_g, E_1_g, and E_2_g modes of the hexagonal phase, consistent with prior reports [50,51,52,53].

#### 3.1.5. Characterization of La_2_O_3_ NPs by XRD

XRD patterns shown in Figure 1E confirmed the crystalline structure of La_2_O_3_ NPs. Diffraction peaks at 2θ = 28.23°, 39.59°, 48.60°, 55.52°, and 64.38° matched ICDD card 01-089-4016, confirming a cubic phase.

#### 3.1.6. Characterization of La_2_O_3_ NPs by TGA

TGA from 0–800 °C shown in Figure 1F revealed thermal stability characteristics [54]. An initial weight loss at 400–500 °C corresponded to decomposition of residual materials. Material above 800 °C corresponded to highly pure La_2_O_3_, free of detectable impurities.

#### 3.1.7. ATR-FTIR of La_2_O_3_ NPs by ATR-FTIR

ATR-FTIR spectra shown in Figure 1G confirmed La–O bonding and identified surface functional groups. Characteristic La–O stretching/bending vibrations were observed below 700 cm^−1^, consistent with La_2_O_3_ metal oxide fingerprints [55,56,57].

Additional bands above 700 cm^−1^ indicated surface functional groups, adsorbed species, and residual precursors. Peaks at 723–827 cm^−1^ corresponded to La–O–C lattice vibrations and carbonate bending, likely from atmospheric CO_2_ adsorption on the hygroscopic surface. The 1043 cm^−1^ band reflected C–O stretching of carbonate/adsorbed CO_2_. Bands at 1388–1498 cm^−1^ indicated CO_3_^2^− asymmetric stretching, suggesting partial surface conversion to La_2_O_2_CO_3_. A 1640 cm^−1^ band from H–O–H bending confirmed adsorbed water. Weak C–H stretches (2850–2925 cm^−1^) likely arose from residual synthesis organics. Broad absorption at 3260–3600 cm^−1^ corresponded to O–H stretching from surface hydroxyls and physisorbed moisture.

Overall, the spectrum confirmed La–O bonding and revealed surface hydroxylation, carbonate formation, and residual adsorbates, features typical of highly reactive, hygroscopic La_2_O_3_ NPs.

#### 3.1.8. Characterization of La_2_O_3_ NPs by Transmission Electron Microscopy

TEM revealed needle-shaped NPs with a mean length of 89.5 ± 41.7 nm and a width of 8.7 ± 2.6 nm. TEM images shown in Figure 1H(a,b) exhibited slender nanoneedles, some aggregating into structures 350–400 nm in length, consistent with prior observations [51,58].

### 3.2. Antibacterial Activity of La_2_O_3_ NPs

**Figure 2:**
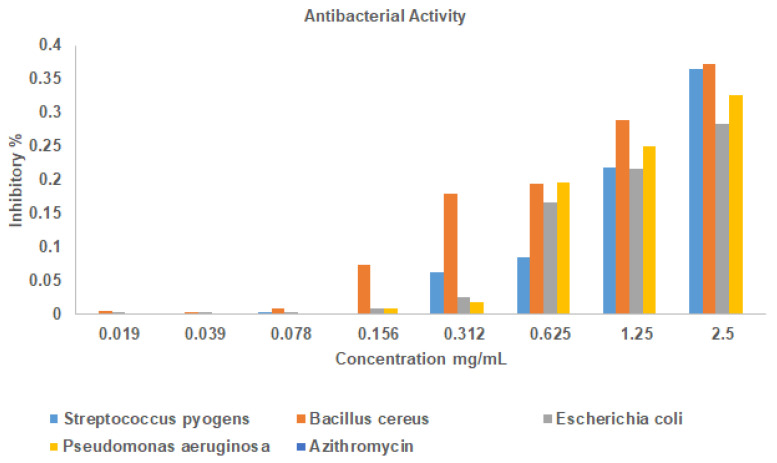
Antibacterial activity of La_2_O_3_ nanoparticles by Resazurin assay.

La_2_O_3_ NPs exhibited only mild antibacterial activity against both Gram-positive and Gram-negative pathogens. Their redox activity may contribute to bactericidal effects, reflected in MIC values of 0.078 mg/L for *Streptococcus pyogenes*, 0.156 mg/L for *Escherichia coli* and *Pseudomonas aeruginosa*, and 0.312 mg/L for *Bacillus cereus*. In addition, it was observed that the La_2_O_3_ NPs tend to sensitize the bacteria, as shown in Figure 2. These effects are likely mediated by multitarget mechanisms, including lipid dephosphorylation, lipid peroxidation, and peptidoglycan disruption [59]. However, when compared with other nanoparticles, La_2_O_3_ shows relatively weak antibacterial potency. For example, silver nanoparticles often demonstrate MIC values near 7 mg/L against *E. coli* [60], while zinc-oxide-based particles frequently require substantially higher concentrations (~64 mg/mL to several hundred mg/mL) depending on the composition and bacterial strain [61]. Thus, La_2_O_3_ NPs fall into a low-potency range. Rather than acting as strong standalone antimicrobials, their significance may lie in multifunctional or synergistic biomedical applications—such as surface coatings, combinational therapies, or scaffolds—where even modest antibacterial activity could complement other biological properties.

### 3.3. Antioxidant Activity of La_2_O_3_ NPs

#### 3.3.1. Antioxidant Activity—Enzymatic Assay Glutathione Reductase Activity

**Figure 3:**
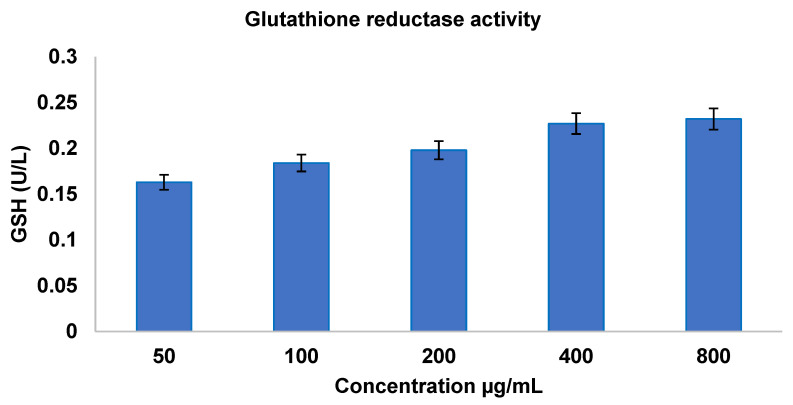
Glutathione reductase antioxidant assay.

Glutathione reductase reduces oxidized glutathione (GSSG) to its thiol form (GSH), thereby scavenging reactive oxygen species. Enzyme activity was measured in La_2_O_3_ NP-treated samples across concentrations (50–800 µg/mL),and is shown in Figure 3.

The activity increased from 0.163 ± 0.06 at 50 µg/mL to 0.232 ± 0.18 at 800 µg/mL before plateauing. Similar dose-dependent enhancements, with distinctions between bulk and nanoscale materials, have been reported previously [62,63].

#### 3.3.2. Antioxidant Activity—Non-Enzymatic Assay 1,1-Diphenyl-2-picrylhydrazyl (DPPH) Assay

**Figure 4:**
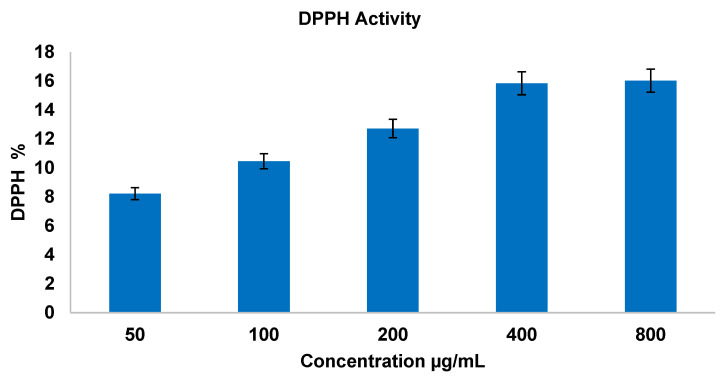
DPPH antioxidant assay.

A similar trend was observed in the DPPH assay, where La_2_O_3_ NPs demonstrated increasing free-radical scavenging, with concentrations shown in Figure 4 [63]. Such antioxidant activity may underlie the differential effects on normal versus cancer cells observed in mammalian assays.

### 3.4. Morphological Evaluation by Optical Microscopy

#### 3.4.1. Cytotoxicity Effect of La_2_O_3_ NPs on Vero Cell Line

**Figure 5:**
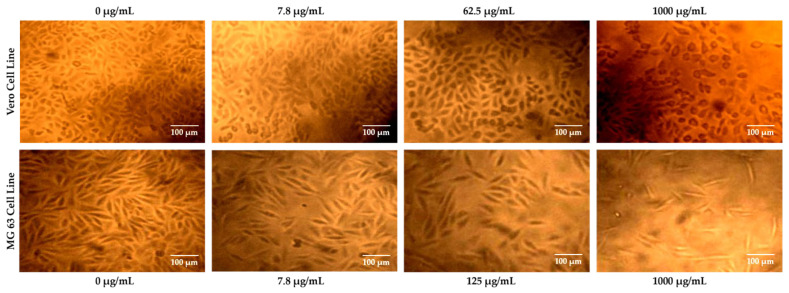
Cytotoxicity effect of La_2_O_3_ NPs on Vero cell line and MG 63 cell line.

**Figure 6:**
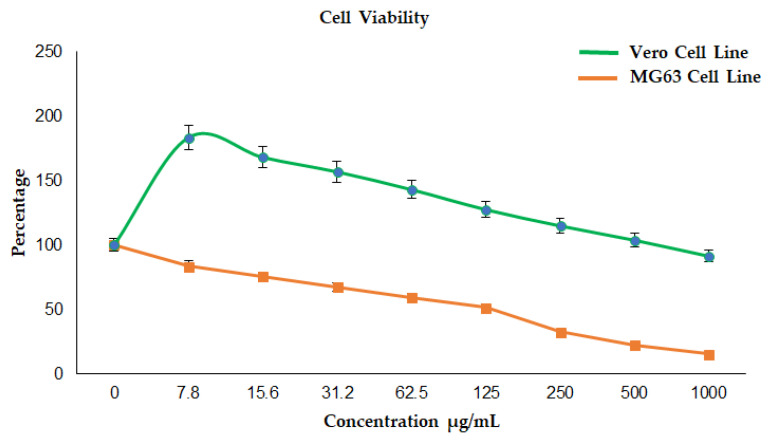
Cell viability of Vero cell line exposed to La_2_O_3_NPs.

Optical microscopy revealed subtle morphological irregularities at low concentrations, progressing to shrinkage, blebbing, and chromatin condensation at higher levels—features consistent with apoptosis, as observed in Figure 5. Low-to-moderate NP concentrations slightly increased Vero cell viability due to its hormetic effect, whereas higher doses reduced viability, as illustrated in Figure 6. Such ROS-mediated morphological changes have been reported in Vero cells under oxidative stress [64].

In Figure 6, it is showing that the increase in concentration of La_2_O_3_NPs leads to a decrease in cell viability in the Vero cell line.

#### 3.4.2. Anticancer Effect of La_2_O_3_ NPs on the MG 63 Cell Line

MG63 viability declined steeply with increasing NP concentration, as illustrated in Figure 5, with more pronounced cell shrinkage, loss of extensions, and organelle damage evident under microscopy. At 125 µg/mL, MG63 viability fell to 51.41%, while Vero viability increased to 127.52%, indicating cancer-selective cytotoxicity. Such selectivity may stem from differences in redox status and NP uptake, as seen in other nanoparticle systems [65].

When plotted together, the viability curves reveal a therapeutic window where MG63 proliferation is suppressed while Vero viability is preserved or enhanced, highlighting selective anticancer potential, as observed in Figure 6. This mirrors observations for Ag NPs, which induce apoptosis in cancer cells while sparing normal cells.

### 3.5. Teratogenicity Assay

**Figure 7:**
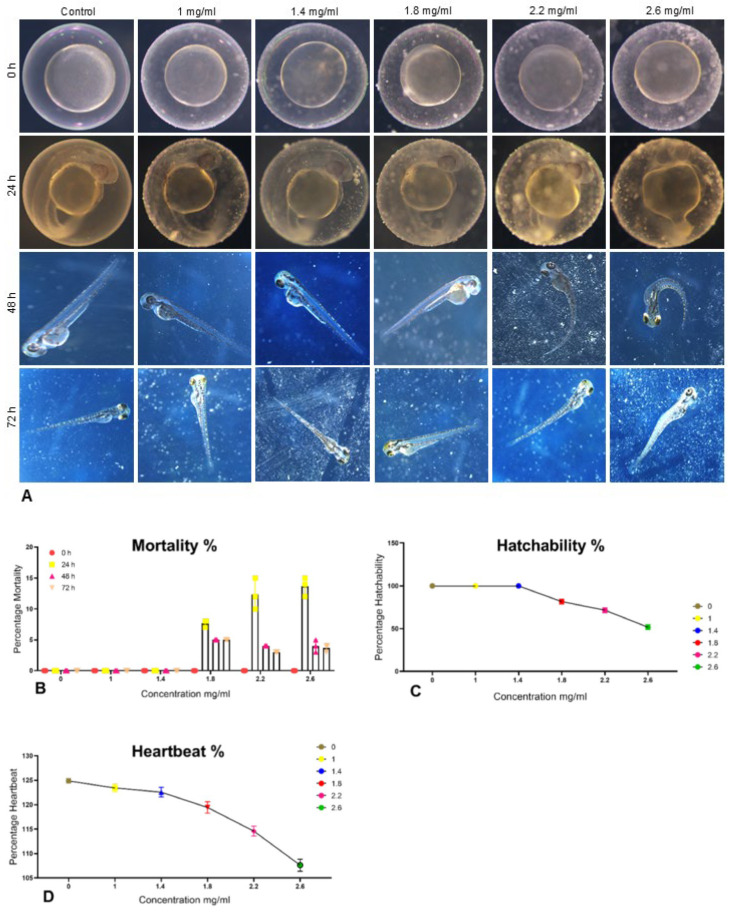
(**A**) Development of zebrafish embryos over time (0–72 h) when exposed to different amounts of La_2_O_3_ NP. (**B**) illustrates the percentage of embryos that died at different La_2_O_3_ NP concentrations; (**C**) depicts the percentage of embryos that hatched successfully; (**D**) shows the impact of La_2_O_3_ NPs on heart rate.

Control embryos showed normal development and 100% hatchability within 48 h. Higher NP concentrations caused somite malformations, tail curvature, reduced hatch rates, and elevated mortality, with LC_50_ determined as 2.6 mg/mL (Figure 7A–D). At 1 mg/mL, no significant adverse effects were noted, indicating a preliminary No Observed Effect Concentration (NOEC).

The LC_50_ value of 2.6 mg/mL for La_2_O_3_ NPs in zebrafish embryos is considerably higher (i.e., less toxic) than that reported for silver nanoparticles (0.03–0.06 mg/mL) [60], but lower than titanium dioxide NPs, which often show negligible mortality up to 10 mg/mL [66]. This positions La_2_O_3_ NPs within an intermediate aquatic toxicity range. Mechanistically, rare-earth ions may disrupt calcium homeostasis and oxygen uptake by blocking chorion pores [67,68], potentially explaining observed developmental abnormalities. ROS generation may further contribute to toxicity, while the NOEC–LC_50_ range defines an initial therapeutic safety margin.

## 4. Conclusions

This study is the first to demonstrate that La_2_O_3_ nanoneedles can simultaneously achieve selective osteosarcoma (MG63) cell cytotoxicity, enhancement of normal cell (Vero) proliferation, potent antibacterial and antioxidant activities, and relatively low developmental toxicity in a vertebrate model. Their distinctive needle-like morphology, cubic crystallinity, and dispersion stability without acidic additives distinguish them from previously reported La_2_O_3_ NPs.

A notable finding was the biphasic (hormetic) response in Vero cells, where low-to-moderate concentrations enhanced viability before cytotoxicity emerged at higher doses. This hormetic profile, combined with the steep dose-dependent cytotoxicity observed in MG63 cells, defines a preliminary therapeutic window for potential biomedical applications. These combined properties position La_2_O_3_ NPs as a multifunctional bioengineering platform with applications in drug-eluting implant coatings, targeted cancer therapeutics, and regenerative medicine scaffolds. Furthermore, the ability to modulate cellular responses in a context-dependent manner opens opportunities for precision biological control.

From a safety perspective, zebrafish embryo assays revealed an LC_50_ of 2.6 mg/mL, placing La_2_O_3_ NPs within an intermediate aquatic toxicity range—less toxic than Ag NPs but more so than TiO_2_—while showing no significant developmental effects at 1 mg/mL. This suggests that lower concentrations may be biologically compatible during early development.

Future work should prioritize in vivo validation, detailed mechanistic studies to dissect the basis of cancer selectivity and hormesis, and optimization of nanoparticle physicochemical parameters (size, shape, and surface chemistry) to maximize efficacy while minimizing environmental and long-term health risks. Such efforts will support the safe, sustainable, and clinically relevant deployment of La_2_O_3_ NPs in advanced biomedical technologies.

## Data Availability

The data used to support the findings of this study are included within the article and are available from the corresponding author upon request.

## Abbreviations

The following abbreviations are used in this manuscript:

MTT	3-(4,5-Dimethylthiazol-2-yl)-2,5-diphenyltetrazolium bromide assay
XRD	X-ray diffractometery
TGA	Thermogravimetric analysis
ATR-FTIR	Attenuated total reflectance Fourier transform infrared spectroscopy
TEM	Transmission electron microscopy
TM	Trademark
NPs	Nanoparticles
BPM	Beats per minute
ATCC	American Type Culture Collection
LC	Lethal concentration
MRI	Magnetic resonance imaging
DNA	Deoxyribonucleic acid
ROS	Radical oxygen species/reactive oxygen species
LAK Cells	Lymphokine-activated killer cells
DMSO	Dimethyl sulphoxide
NCCS	National Centre for Cell Sciences
UK	United Kingdom
EDTA	Ethylenediaminetetraacetic acid
GSSG	Glutathione disulfide
NADPH	Nicotinamide Adenine Dinucleotide Phosphate Hydrogen
UV	Ultraviolet
GSH	Reduced glutathione
DPPH	1,1-diphenyl-2-picrylhydrazyl
BHT	Butylated Hydroxy Toluene
RSA	Radical scavenging activity
DMEM	Dulbecco’s Modified Eagle’s Medium
IC	Inhibitory concentration
CCSEA	Committee for the Control and Supervision of Experiments on Animals
OECD	Organisation for Economic Co-operation and Development
ICDD	International Centre for Diffraction Data
OD	Outer diameter/optical density
MIC	Minimum inhibitory concentration
OM	Optical microscope
FBS	Foetal bovine serum
NOEC	No Observed Effect Concentration
